# Characteristics of house calls in Brazil: analysis of PMAQ-AB external evaluation cycles

**DOI:** 10.1590/1980-549720240007

**Published:** 2024-02-26

**Authors:** Luan Henrique Honório Rocha, Ana Graziela Araujo Ribeiro, Vanessa Almeida Silva, Francenilde Silva de Sousa, Erika Barbara Abreu Fonseca Thomaz

**Affiliations:** I Universidade Federal do Maranhão, Department of Medicine - São Luís (MA), Brazil.; II Universidade Federal do Maranhão, Postgraduate Program in Public Health - São Luís (MA), Brazil.

**Keywords:** House calls, Health services, Health evaluation, Primary health care, Visita domiciliar, Serviços de saúde, Avaliação em saúde, Atenção primária à saúde

## Abstract

**Objective:**

To analyze characteristics of the home visit (HV) in Brazil, 2012 and 2017.

**Methods:**

Ecological study, with panel data whose units of analysis were the Primary Health Care teams in Brazil, participants of the 1st and 3rd cycles of the Program to Improve Access and Quality of Primary Care of the Unified Health System. Descriptive, inferential and spatial analyzes (alpha=5%) were performed.

**Results:**

There was an increase in the proportion of teams that carried out home visits at a frequency defined based on risk and vulnerability analysis and actively searched for people with respiratory symptoms and women with delayed and altered cytopathological examination. In the heat maps, the Northeast, Southeast and South regions had a higher concentration of teams that carried out the HV and carried out an active search.

**Conclusion:**

The maintenance and qualification of HVs must be a priority in Brazil, since there are few countries in the world with such capillarity of health services, reaching the homes of millions of people.

## RESUMO

**Objetivo:**

Analisar características da visita domiciliar (VD) no Brasil em 2012 e 2017.

**Métodos:**

Estudo ecológico com dados em painel cujas unidades de análise foram as equipes de saúde da Atenção Primária à Saúde no Brasil, participantes do 1º e 3º ciclos do Programa de Melhoria do Acesso e Qualidade da Atenção Básica do Sistema Único de Saúde. Foram realizadas análises descritivas, inferenciais e espaciais (alpha=5%).

**Resultados:**

Houve aumento na proporção de equipes que realizavam visita domiciliar com periodicidade definida a partir da análise de risco e vulnerabilidade e na de busca ativa de pessoas com sintomáticos respiratórios e de mulheres com exame citopatológico atrasado e alterado. Nordeste, Sudeste e Sul foram as regiões com maior concentração de equipes que realizavam a VD e faziam busca ativa.

**Conclusão:**

A manutenção e qualificação das VD deve ser uma prioridade no Brasil, uma vez que poucos são os países no mundo com tamanha capilaridade dos serviços de saúde, alcançando os domicílios de milhões de pessoas.

## INTRODUCTION

The Family Health Strategy (FHS), designed in 1994 as the Family Health Program (FHP), is one of the strongest actions for consolidating Primary Health Care (PHC), with the objective of promoting, preventing, recovering, and rehabilitating users’ health, with an emphasis on family as the central nucleus of care. Among the services offered that contribute to achieving its objectives, there is House Calls (HC)^1^ ^,^ ^2^.

HC allows health care to be provided in a more humane and welcoming way, bringing professionals closer to the community, creating an emotional bond between the parties involved and expanding the population’s access to health actions at one of the points in their care network: the home of each family. In this way, it is possible to achieve greater care effectiveness of both individuals and their families^3^. Therefore, HC are of great importance as they allow professionals to provide assistance, collect data on housing conditions, and apply measures to control communicable or parasitic diseases, contributing to health education and community empowerment^4^.

In the international scenario, house calls have a similar meaning to that in Brazil, providing health care to users and family members, allowing professionals to get closer to the community. In fact, there are studies that describe specific care offered during these visits in other countries, such as in the diet of users, incentives to improve the physical and psychosocial health of the aged, and postnatal care for women who have recently given birth^5^ ^,^ ^6^ ^,^ ^7^.

In Brazil, there is evidence that in places where HC are carried out properly, there are higher rates of early initiation of prenatal consultations, six or more prenatal consultations, vaccinations, testing, vitamin supplementation, and guidance for pregnant women, exclusive breastfeeding until six months of the baby’s life^8^ ^,^ ^9^ ^,^ ^10^ ^,^ ^11^, in addition to increasing user satisfaction with PHC^10^ and reducing hospital admissions^11^ ^,^ ^12^. The visits reduce women’s doubts, fears, and anxieties regarding prenatal care, childbirth, the postpartum period, and child care. The presence of nurses and doctors in HC reduces the prevalence of problems related to psychological distress and qualifies physical examinations of women and children^11^ ^,^ ^13^.

However, a tool capable of organizing the professionals’ work process during home visits has not yet been developed/implemented in PHC services in Brazil. Therefore, there may be different HC formats, and a protocol could support these professionals. In some teams, HC can only be carried out by the Community Health Worker (CHW), while others include more team professionals. Visits may also differ regarding the use or not of protocols that take into account the vulnerability of people in the territory, as well as the use or not of planning and monitoring tools for users^14^ ^,^ ^15^ ^,^ ^16^.

Therefore, given the lack of a quality standard for HC in Brazil, the way these visits have been carried out in the daily routine of home care services is unknown. The studies identified are restricted to some municipalities in the states, simulating controlled HC conditions in community trials, or are literature reviews, with historical analysis of home visiting practices in the context before and after the Brazilian Unified Health System (*Sistema Único de Saúde* - SUS)^10^ ^,^ ^11^ ^,^ ^17^ ^,^ ^18^.

The studies identified do not analyze characteristics related to HC protocols and the profile of the professionals involved, nor the use of action planning tools according to the vulnerability of users^9^ ^,^ ^10^ ^,^ ^11^. Therefore, this study analyzed the characteristics of HC in Brazil, in two moments: 2012 and 2017.

## METHODS

This is an ecological study, with panel data and a spatio-temporal approach. The units analyzed were the Family Health and Parameterized teams (we standardized the term PHC health teams) in Brazil, participants in the 1^st^ and 3^rd^ cycles of the Program for Improving Access and Quality of Primary Care of the Unified Health System (*Programa de Melhoria do Acesso e Qualidade da Atenção Básica do Sistema Único de Saúde* - PMAQ-AB). For spatial analyses, data were aggregated to the level of health regions. The study was written according to STROBE recommendations^19^.

The first cycle of PMAQ-AB took place in 2012, simultaneously with UBS census, in which the infrastructure, equipment, facilities, human and material resources of these units were examined. The second and third cycles of the program, carried out in 2014 and 2017, respectively, were aimed at health units and teams that joined the program^20^ ^,^ ^21^.

PMAQ-AB assessments were commissioned by the Ministry of Health and conducted by a consortium formed by several Brazilian universities and research centers. The fieldwork was carried out by health professionals and evaluators trained by the Ministry of Health. The evaluation instrument used was organized into six modules structured within four major aspects: organization of services; work management, health care; and user satisfaction^22^ ^,^ ^23^.

For this study, data from the 1^st^ and 3^rd^ cycles were considered regarding the work process of PHC health teams that could be associated with HC (module 2). The microdata were obtained from the Ministry of Health databases, available to the public electronically: http://aps.saude.gov.br/ape/pmaq. To create the maps, data from the Information System of the Brazilian Institute of Geography and Statistics (*Instituto Brasileiro de Geografia e Estatística* - IBGE) were used.

From PMAQ-AB, the following variables were considered:

1. Does the team carry out house calls?;
2. Do professionals other than CHWs participate?;
3. Does the team actively search for postpartum women?;
4. Does the team actively search for respiratory symptoms?;
5. Does the team actively search for pregnant women, those with high blood pressure, and absenteeism?;
6. Does the team actively search for women with delayed cytopathological examination?;
7. Does the team actively search for women with altered cytopathology?;
8. Does the team carry out home visits at defined intervals based on risk and vulnerability analysis?;
9. Does the team organize the demand for house calls?

The possible answers were yes or no to all questions.

Some questions required adaptations to promote compatibility between the variables of both cycles. In cycle 3, a team that did not carry out HC was considered to be one that responded “does not make house calls” to the question “does the team carry out house calls at different intervals?”. In cycle 1, the questions involving active search in the territory of diabetics, hypertensive patients, and pregnant women who were absent were grouped, corresponding to the question “carrying out an active search for those who were absent or abandoning treatment” in cycle 3. The “organization of demand for house calls” was the question evaluated in the third cycle, comparing with the first through the question “does the team have a survey/mapping of registered users who need to receive care at home?”.

Descriptive and inferential statistical analyses were carried out, estimating absolute frequencies, percentages, and confidence intervals for categorical variables, as well as means and standard deviations for numerical variables.

Spatial analysis was also carried out to describe the spatial distribution of variables in the federative units for both PMAQ-AB cycles. In this analysis, spatial aggregation was performed by municipality (proportion of adequate responses per municipality) and summarized using averages. Choroplectic maps were created, whose variables were classified on a 5-level scale: 0-24.99%; 25-49.99%; 50-74.99%; 75-100%; and without information (municipalities that did not have information for the variable).

Kernel maps were created. This is a simplified method to estimate the intensity of a phenomenon in a given area using Kernel density. This way, it is possible to have an overview of the intensity of the analyzed variable in the regions of the map. The variables were also classified on a 5-level scale (very low, low, medium, high, and very high), ranging from colder to warmer colors, depending on the degree of intensity of the phenomenon^24^.

The analyses were carried out using Stata^®^ (version 14) and QGIS^®^ (version 3.16.9). Absolute and relative frequencies were presented, accompanied by their respective 95% confidence intervals (95%CI). The study was approved by the Research Ethics Committee of the University Hospital of Universidade Federal do Maranhão (CEP HUUFMA), under CAAE No. 92281818.9.1001.5086.

## RESULTS

There were 17,203 PHC health teams participating in the first cycle and 38,865 teams in the third. More than 98% of the teams reported carrying out HC in both cycles. There was an increase in the proportion of teams that carried out HC at a frequency defined based on risk and vulnerability analysis, from 92.9% (92.5-93.3) to 98.4% (98.2-98.5). There was also an increase in the proportion of teams that actively searched for people with respiratory symptoms, from 77.7% (77.1-78.3) to 79.9% (79.5-80.3), and women with delayed and altered cytopathological examination, from 75.7% (75.1-76.4) to 86.2% (85.8-86.5) and from 86.7% (86.1-87.2) to 96.8% (96.6-97.0), respectively (Table 1).

**Table 1.** Changes in house calls characteristics between PMAQ-AB cycles 1 and 3. Brazil, 2012 and 2017.

Characteristics of house calls	Cycle 1 (17,203 PHC health teams)			Cycle 3 (38,865 PHC health teams)		
	n*	%^†^	IC95%^‡^	n*	%^†^	IC95%^‡^
The team carries out house calls	17,132	99.6	99.5-99.7	36,702	98.3	98.1-98.4
In addition to CHW, other professionals participate in the visit	14,214	82.6	82.0-83.2	29,025	77.7	77.3-78.1
The team actively searches for pregnant women, those with high blood pressure, and people who are absent	16,537	96.1	95.8-96.4	29,855	79.9	79.5-80.3
The team actively searches for respiratory symptoms	13,370	77.7	77.1-78.3	29,833	79.9	79.5-80.3
The team actively searches for postpartum women	15,447	89.8	89.3-90.2	33,391	89.4	89.1-89.7
The team actively searches for women with delayed cytopathological examination	13,026	75.7	75.1-76.4	32,199	86.2	85.8-86.5
The team actively searches for women with altered cytopathological examination	14,909	86.7	86.1-87.2	36,149	96.8	96.6-97.0
The team carries out house calls at defined intervals based on risk and vulnerability analysis	15,981	92.9	92.5-93.3	36,739	98.4	98.2-98.5
The team organizes the demand for home care	12,105	70.4	69.7-71.0	24,967	66.8	66.4-67.3

*Absolute number of PHC health teams; ^†^Percentage of PHC health teams; ^‡^95% confidence interval. PHC: Primary Health Care.

On the other hand, there was a reduction in the proportion of teams that actively searched for pregnant women, hypertensive patients and absentees from 96.1% (95.8-96.4) to 79.93% (79.5-80.3). Other professionals, in addition to CHWs, participated in these visits in 82.6% (82.0-83.2) of the teams in cycle 1, decreasing to 77.7% (77.3-78.1) in cycle 3 (Table 1). 

**Figure 1.**
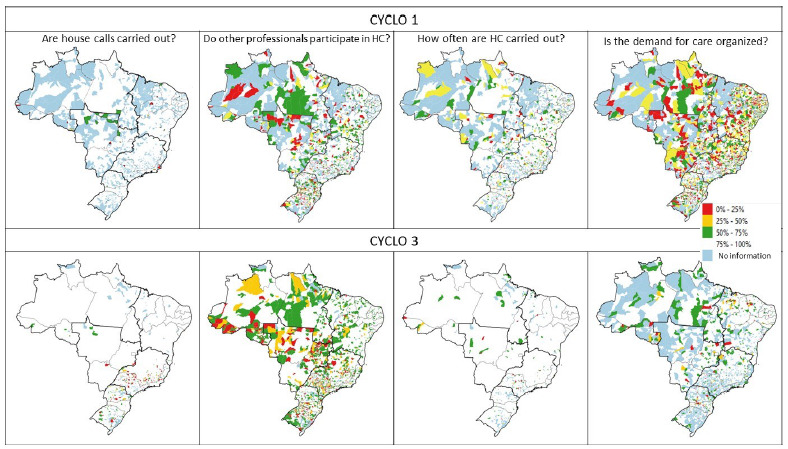
Choropleth maps. House calls. PMAQ-AB 1st and 3rd cycles (by municipality), 2021.

**Figure 2.**
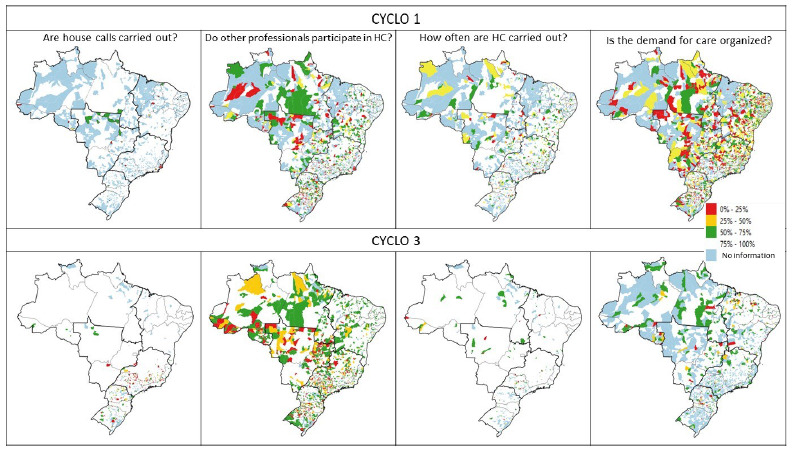
Choropleth maps. Active search. PMAQ-AB 1st and 3rd cycles (by municipality), 2021.

AS: active search; HBP: high blood pressure.

Choropleth maps demonstrate an improvement in the spatial distribution of variables related to HC and active search between cycles 1 and 3 in Brazilian regions and for most federative units, suggesting progress in the performance and quality of HC (Figures 1 and 2).

HC: house calls

Although there was a significant increase in teams that include professionals other than CHWs in HC in cycle 3 (white areas of the map), areas that remain with low percentages of this variable were identified. The Northeast region has the best performance, with practically the entire territory having at least 50% of teams with HCs performed by more than one professional (Figure 1).

**Figure 3.**
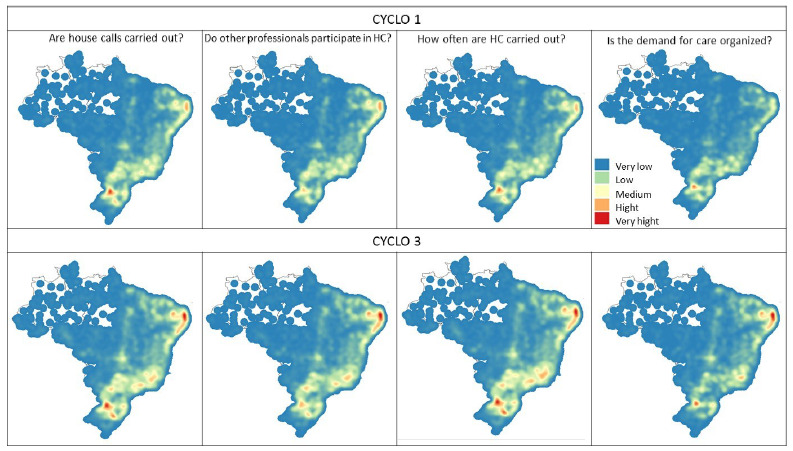
Heatmaps (Kernel). House calls. PMAQ-AB 1st and 3rd cycles (by municipalities), 2021.

**Figure 4.**
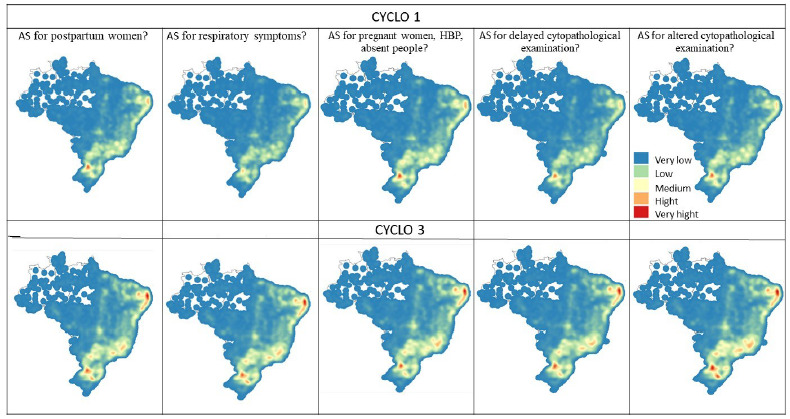
Heatmaps (Kernel). Active search. PMAQ-AB 1st and 3rd cycles (by municipality), 2021.

Figures 3 and 4 represent the heat maps for each variable in cycles 1 and 3. In the images, it can be seen that the Northeast, Southeast, and South regions have a greater concentration of teams that carry out the action (variable) analyzed, both for cycles 1 and 3. For cycle 3, a greater concentration of teams was identified in the states of Rio Grande do Norte, Paraíba, Pernambuco, and Alagoas, as well as in the states of the Southeast and South regions of the country, carrying out the actions evaluated for HC. The other Brazilian regions had a very low concentration of teams carrying out the actions investigated (Figures 3 and 4).

## DISCUSSION

The performance, frequency, and organization of HCs improved in Brazil, comparing cycles 1 and 3 of the PMAQ-AB. The active search for women with delayed or altered cytopathological examination and respiratory symptoms improved in the North, Northeast, and Central-West regions. However, the active search for pregnant women, hypertensive patients, and absentees worsened between assessments.

Visits allow health professionals to get to know the reality of users, families, and the community up close, in order to enable these professionals to identify health needs and demands, establish bonds of trust, and promote people’s active participation in the care process. Thus, this service can be highlighted as relevant for longitudinality, comprehensiveness, and coordination of care, leading to the strengthening of PHC^25^.

Furthermore, visits must be carried out based on risk and vulnerability criteria, so that families with greater needs are visited more often. Considering that these are people in need of more intense monitoring, it is important that the frequency of visits to this public exceeds the average recommended in the National Primary Care Policy (*Política Nacional de Atenção Básica* - PNAB)^26^. In view of this, it is important that teams know how to carry out a family risk stratification assessment, using scales such as the Coelho Savassi Family Risk Scale^27^.

Although PNAB 2017 establishes the average monthly frequency of CHW visits to be carried out in each household, it does not define the criteria to their frequency. Hence, it is worth noting possible difficulties in standardizing and prioritizing HC, whether carried out exclusively by CHWs or jointly with other team professionals^26^.

An increase in the proportion of HC was observed between cycles, however, some teams continue not to do so, especially in the South and Southeast of the country. Work overload is reported by agents as difficulty in carrying out visits^28^, signaling the teams’ lack of clarity regarding the CHW’s main duties, displacing them to other activities that require time and make it difficult to carry out HC as recommended, or even more frequently, if necessary^26^.

This situation has also directly reflected on the perception of families and other professionals, given that users and nurses were dissatisfied with the low number of families visited by CHWs^29^. A meta-synthesis on the work of the CHWs in FHS identified articles in which the theme of “lack of sizing of the work of CHWs” was addressed, highlighting the excess of expected functions and the lack of clarity of their responsibilities^30^.

In a study carried out in the Southeast region of Brazil, Costa and collaborators showed that 33.6% of CHWs reported helping higher-level professionals in clinical environments, taking on tasks such as disinfection and sterilization of clinical instruments, which would be the responsibility of nursing technicians or assistants, or administrative functions, such as scheduling appointments and exams and checking the stock of office and medical-hospital supplies. Situations like these, in addition to diverting the focus of actions in the territory, mischaracterize the role of CHWs in the health team and make it difficult to consolidate their own professional identity^29^.

It is noticed that fewer health professionals, in addition to CHWs, are carrying out HC in Brazil. This may explain why research is increasingly addressing the issue of work overload that CHWs experience, since they work in different roles, have very broad responsibilities, and are increasingly alone during HC^28^ ^,^ ^31^.

In addition to this aspect addressed by CHWs themselves, it is important to address the possible impacts of the review of PNAB, published in 2017, on HC. PNAB became the target of criticism due to restrictive measures that put the organization of PHC at risk^32^. One of the most prominent problematized measures was the flexibility of the presence of CHWs in the composition of FHS, which could lead to the discontinuity of actions focused on the territory, compromising access and effectiveness of health actions^33^. This could have potential long-term negative impacts on both HC and other services such as vaccinations and consultations^32^.

This study also showed that the active search for pregnant women, hypertensive patients, and absentees worsened between assessments. In the last evaluation, the states of Acre, Rondônia, and Mato Grosso presented proportions below 25%. It was also observed that the Southeast and South regions had the worst rates, when compared to the other regions of the country.

Monitoring women must be carried out in a welcoming manner and must occur since prenatal care, to promote the correct provision of care to the mother-baby binomial^13^. Actively searching for pregnant women positively influences adherence to prenatal care, which leads to a better outcome during childbirth and increases the chances of women maintaining the bond during the postpartum period. It can also reduce the occurrence of new unplanned pregnancies and adherence to better self-care and baby care practices. Therefore, this active search with very low values reveals a serious public health problem in these states.

These results may reflect a lack of structure to carry out HC, such as insufficient CHWs, problems in monitoring these conditions by PHC professionals, problems with transportation from the health unit to the home of the family to be visited, problems with acceptance of visits by the community, among other factors. Also noteworthy are the FHS’s difficulties in the continued management of families, the invasion of FHS territories, the persistent culture of referral and health responsibility centered on nurses, which can make it difficult to carry out active search^34^.

In relation to improving the active search for delayed and/or altered cytopathology in Brazil, it is known that the Pap smear is a simple procedure that allows finding changes in the uterine cervix, being the most appropriate method for screening cervical cancer as a quick, painless, easy-to-perform exam, carried out at primary care level, in addition to its low cost^35^. When early diagnosed, the possibility of curing cervical cancer is 100%. However, as the proportion of women who do not routinely undergo preventive examinations is still high, diagnosis is often still made at a more advanced stage of the disease^36^. Amapá stood out as a state whose indicators worsened between cycles.

According to the Ministry of Health, those most “responsible” for the high levels of cervical cancer and the non-acceptance of the Pap smear in Brazil are: lack of human resources and inputs released into the health network for prevention, diagnosis, and treatment; inadequate application of living resources; poor connection between health services in providing assistance at different levels of care; inaccuracy of standards and conduct; lack of health information for the general population; and lack of clarification necessary for planning health actions^35^ ^,^ ^36^.

The active search for respiratory symptoms has improved over the years, observed when comparing cycles, and the creation of the National Tuberculosis Control Program (*Programa Nacional do Controle da Tuberculose* - PNCT) was one of the factors that had a positive impact on Brazilian indicators related to tuberculosis, as it aims to “horizontalize” the fight against tuberculosis, through the expansion of its activities to all SUS health services. All national plans and consensuses for tuberculosis control that followed emphasized its integration into basic care, using FHS as a way to expand access to diagnosis and treatment of tuberculosis throughout Brazil^37^.

In general, a significant increase in the number of teams that participated in the PMAQ-AB between cycles 1 and 3 was noted. In general, participants in cycle 1 were the establishments with the best organized teams. However, in the last cycle, almost all teams were evaluated, enabling a more realistic analysis of the situation experienced by CHWs in Brazil. In addition to being a strong point of this study, it also makes it possible to think that the improvement in active search indicators for women with delayed or altered cytopathology and respiratory symptoms was actually greater than what was found, showing real advances in active search.

This study used data from the external evaluation of two PMAQ-AB cycles, carried out in 2012 and 2017, therefore, prior to the COVID-19 pandemic. This can be considered a limitation of the study, given that the pandemic forced a reorganization of health systems, which caused the suspension or reorganization of HC across the country^38^.

HC were greatly affected during the pandemic, as receiving professionals in users’ homes was no longer a recommended practice. In addition to the lack of protocols for the restructuring of PHC in the context of the pandemic as a major issue, many changes were highlighted during this period, such as the recommendation for visits to be carried out in an external environment, active search via WhatsApp or telephone, and the use of personal protective equipment (PPE) during HC^39^ ^,^ ^40^.

However, the results of this study demonstrate possibilities of regions and paths that can serve as a mirror for the reorganization of services in PHC. For example, we realized that CHWs could have been more active professionals in containing the pandemic, being a powerful option for: early identification of infected patients; possibility of monitoring the care of infected patients outside the hospital; guidance to family members on the importance of using social isolation measures, wearing a mask and vaccination; continuity of care for people with chronic illnesses^41^.

Despite the advances noticed between PMAQ-AB cycles in terms of carrying out, frequency, and organization of HC, it is possible that visits are taking place in a very restricted manner, or even not being carried out in some places due to the pandemic.

One may then realize that this is, in fact, a very important tool for tackling health problems in populations. There are few countries in the world with such capillarity of health services, reaching the homes of millions of people, therefore, the maintenance and qualification of HC must be a priority, even in the face of pandemics.
